# Genotypic Variation in Growth and Physiological Response to Drought Stress and Re-Watering Reveals the Critical Role of Recovery in Drought Adaptation in Maize Seedlings

**DOI:** 10.3389/fpls.2015.01241

**Published:** 2016-01-12

**Authors:** Daoqian Chen, Shiwen Wang, Beibei Cao, Dan Cao, Guohui Leng, Hongbing Li, Lina Yin, Lun Shan, Xiping Deng

**Affiliations:** ^1^State Key Laboratory of Soil Erosion and Dryland Farming on the Loess Plateau, Institute of Soil and Water Conservation, Northwest A&F UniversityYangling, China; ^2^College of Life Sciences, Northwest A&F UniversityYangling, China; ^3^College of Natural Resources and Environment, Northwest A&F UniversityYangling, China; ^4^Institute of Soil and Water Conservation, Chinese Academy of Sciences and Ministry of Water ResourcesYangling, China; ^5^Beijing Zhongnong Chuangyou Seed Technology Corporation LimitedBeijing, China

**Keywords:** drought adaptation, genotypic variation, drought resistance, drought recovery, maize

## Abstract

Non-irrigated crops in temperate climates and irrigated crops in arid climates are subjected to continuous cycles of water stress and re-watering. Thus, fast and efficient recovery from water stress may be among the key determinants of plant drought adaptation. The present study was designed to comparatively analyze the roles of drought resistance and drought recovery in drought adaptation and to investigate the physiological basis of genotypic variation in drought adaptation in maize (*Zea mays*) seedlings. As the seedlings behavior in growth associate with yield under drought, it could partly reflect the potential of drought adaptability. Growth and physiological responses to progressive drought stress and recovery were observed in seedlings of 10 maize lines. The results showed that drought adaptability is closely related to drought recovery (*r* = 0.714^**^), but not to drought resistance (*r* = 0.332). Drought induced decreases in leaf water content, water potential, osmotic potential, gas exchange parameters, chlorophyll content, Fv/Fm and nitrogen content, and increased H_2_O_2_ accumulation and lipid peroxidation. After recovery, most of these physiological parameters rapidly returned to normal levels. The physiological responses varied between lines. Further correlation analysis indicated that the physiological bases of drought resistance and drought recovery are definitely different, and that maintaining higher chlorophyll content (*r* = 0.874^***^) and Fv/Fm (*r* = 0.626^*^) under drought stress contributes to drought recovery. Our results suggest that both drought resistance and recovery are key determinants of plant drought adaptation, and that drought recovery may play a more important role than previously thought. In addition, leaf water potential, chlorophyll content and Fv/Fm could be used as efficient reference indicators in the selection of drought-adaptive genotypes.

## Introduction

Drought stress imposes huge reductions in crop yield and is one of the greatest limitations to crop production outside present-day agriculture areas (Chaves et al., 2009). Because predictions of future global environmental change involve increases in the both severity and frequency of drought in the near future (Dai, 2013), engineering and breeding more efficient and better drought adapted crop cultivars is becoming increasingly important. Understanding the mechanisms of drought response and adaption is fundamental for the achievement of those goals.

Plants have evolved several different types of drought-adaptive strategies which allow them to adapt to specific habitats for the benefit of their growth and development (Fang and Xiong, 2015). Drought resistance has been a major concern in plant drought adaptation (Verslues and Juenger, 2011; Fang and Xiong, 2015; Kooyers, 2015). Generally, plant drought resistance involves drought escape via a short life cycle or developmental plasticity (Manavalan et al., 2009), drought avoidance via enhanced water uptake and reduced water loss (Luo, 2010; Tardieu, 2013), or drought tolerance via osmotic adjustment, antioxidant capacity, and desiccation tolerance (Yue et al., 2006; Luo, 2010). The molecular and physiological mechanisms associated with drought resistance have been extensively studied and is being elucidated, especially in the model plants like Arabidopsis (Wei et al., 2009; Golldack et al., 2014; Hu and Xiong, 2014; Nakashima et al., 2014; Osakabe et al., 2014; Yoshida et al., 2014; Todaka et al., 2015). In Arabidopsis, drought resistance is often measured as survival. Drought resistance is defined by agronomists in terms of “relative yield of genotypes” or “the ability of a crop plant to produce its economic product with minimum loss in a water-deficit environment relative to the water-constraint free management” (Fukai and Cooper, 1995; Fang and Xiong, 2015). Yield is a complex trait, and yield under drought (the definition of drought resistance in crop plants) is polygenic and has low heritability, hence it is difficult to select (Turner et al., 2014). As yield is determined by growth and developmental processes, plant growth was considered as a measure of environmental input and adaptive capacity to a particular environment (Blum, 1979; Dolferus, 2014). Therefore, in crop species like cereals, maintenance of growth under drought is more important than survival (Dolferus, 2014).

In terms of practical agriculture, yield loss is unavoidable under incessant drought conditions. Non-irrigated crops in temperate climates and irrigated crops under arid climates are subjected to continuous cycles of water stress and re-watering (Perrone et al., 2012). When water shortage is relieved, the crops need to restart growth as quickly as possible. Recovery after stress is a very complex process involving the rearrangement of many metabolic pathways to repair drought-induced damage and to resume plant growth. It requires far more than simply a return to the state before stress onset (Vanková et al., 2012). A plant's capability to resume growth and gain yield after exposure to severe drought stress which causes a complete loss of turgor pressure and leaf dehydration has been defined as drought recovery (Luo, 2010; Fang and Xiong, 2015). Drought adaptability is defined as the comprehensive capacity integrating both drought resistance and recovery for adaptation to drought stress and re-watering. While drought resistance, especially drought avoidance and tolerance, has been a major concern in previous studies on plant drought adaptation, the role of drought recovery in plant drought adaptation has received much less attention over the last several decades. Recently, increasing importance has been attached to drought recovery in crops (Chaves et al., 2009; Luo, 2010; Perrone et al., 2012; Vanková et al., 2012; Fang and Xiong, 2015). Luo (2010) proposed that drought recovery is involved in drought resistance in crops. Fang and Xiong (2015) regard drought recovery as a major component of drought resistance, along with drought avoidance, drought tolerance and drought escape.

Plants respond and adapt to drought stress through the induction of various morphological and physiological responses (Wang and Huang, 2004). Many physiological factors may be involved in drought stress injury (Jiang and Huang, 2001); for example, drought stress both damages the photosynthetic apparatus and diminishes chlorophyll content (Fu and Huang, 2001). The multiplicity of factors involved in drought stress injury suggests that screening studies of many kinds may be useful for characterizing drought resistance (Hura et al., 2007). Field trials of breeding-stock plants with regard to their capacity for natural drought adaptation are time-consuming and expensive due to limited space and available seed of certain genotypes, especially in early generations. More importantly, the severity of natural drought stress is uncontrollable in field conditions. Therefore, there is an urgent need to find affordable and trustworthy physiological indicators that can help in the selection of drought-adaptive genotypes (Hura et al., 2007). However, previous studies in this area have generally focused on drought resistance and ignored drought recovery.

Yield is determined by growth and developmental processes and plant growth was considered as a measure of drought adaptive capacity (Blum, 1979; Dolferus, 2014). As the seedlings behavior in growth associate with yield under drought, it could partly reflect the potential of drought adaptability (Liu et al., 2013; Ramegowda et al., 2014; Yang et al., 2014). In the present study, the roles of drought resistance and drought recovery in maize drought adaptation were comparatively analyze based on the seedling relative growth before field test along with yield. The seedlings of 10 maize (*Zea mays*) lines were subjected to a progressive soil drought and subsequent re-watering treatment. Growth was measured during drought stress and re-watering cycle. Drought resistance, drought recovery and drought adaptability were estimated based on the relative growth. Furthermore, several physiological traits, including the relative water content, water potential, osmotic potential, gas exchange parameters, chlorophyll content, chlorophyll fluorescence, H_2_O_2_ content, lipid peroxidation, carbohydrate content, nitrogen content and C/N ratio were assessed to investigate the physiological basis of genotypic variation in drought adaptation and to characterize the physiological traits that may indicate plant capacity for drought recovery and drought adaptability.

## Materials and methods

### Plant growth conditions and drought treatments

**Figure 1:**
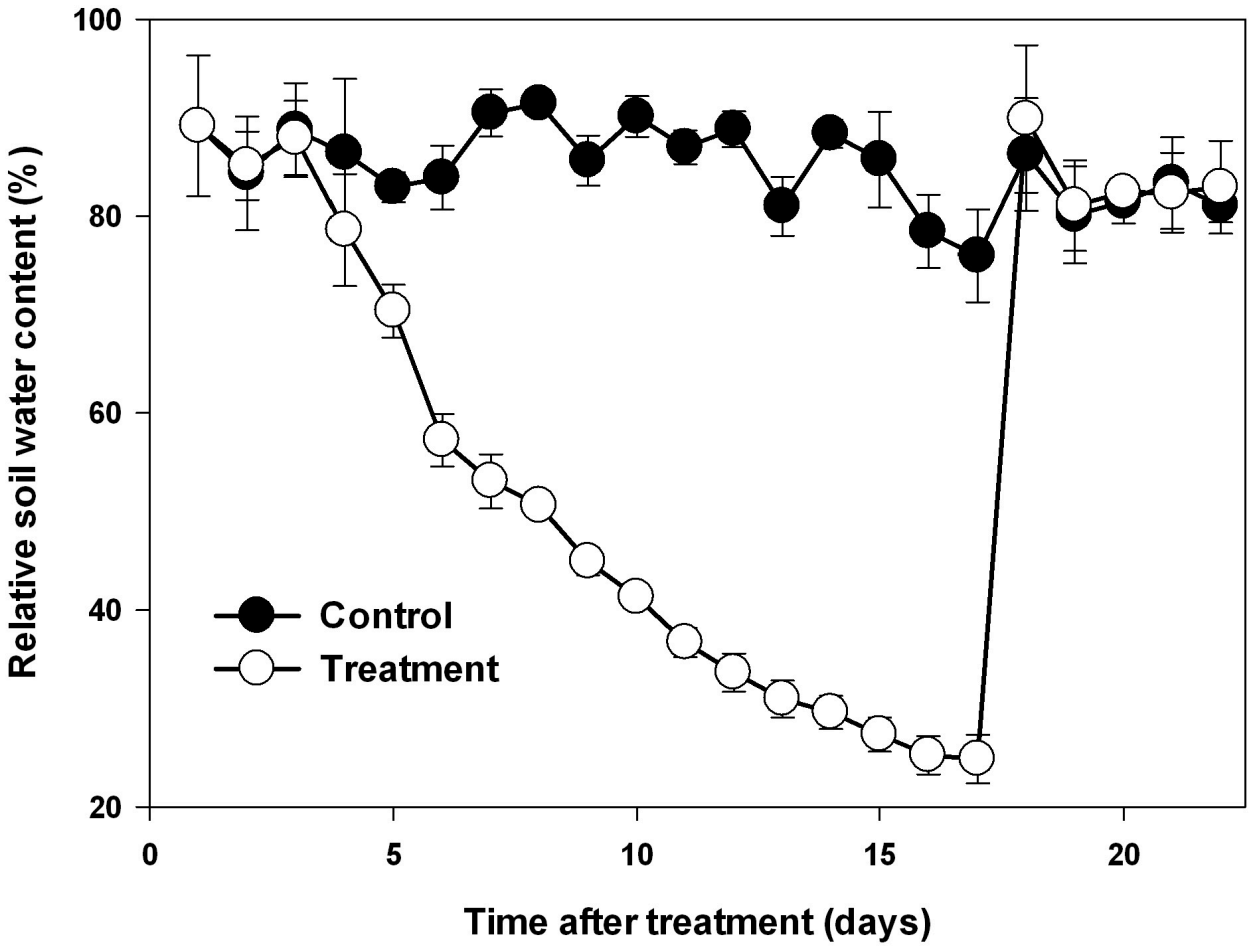
**Changes in soil water content during drought stress and re-watering. Data represent the mean ± SD (*n* = 30)**.

Nine maize inbred lines (L1~6, L8~10) and the hybrid line Zhengdan 958 (L7), one of the most popular cultivar of maize in China in terms of total area planted, were used in this study. The experiment was conducted in a greenhouse at the Institute of Soil and Water Conservation, Northwest A&F University (altitude of 530 m, 34°120′N, 108° 70′E) with a natural photoperiod (approximately 14 h light) from April to June 2014. The daily mean air temperature during the experiment is shown in Supplementary Figure 1. The seeds were sterilized with 1% sodium hypochlorite for 10 min and then washed with distilled water four times. After sterilization, six seeds were sown per plastic pot (Diameter × Height: 300 × 290 mm) filled with 15 kg loessial soil collected from the upper 20 cm of a cultivated field. The seedlings were thinned to two seedlings per pot when the third leaves were fully expanded. When the eighth leaves were fully expanded in a majority of the lines, a natural progressive drought was imposed by withholding watering based on daily measurements of pot weight. Soil water content was calculated according to the weight and expressed as a percent maximum pot capacity (Chen et al., 2015). In the control pots, soil water content was maintained between 80 and 90% throughout the experiment; in the treatment pots, the water content of the soil was allowed to fall progressively for 17 days, with daily water supplement to keep all pots consistency, and was then restored to the control level and maintained for 5 days, as shown in Figure 1. The above-ground biomass was harvested at the start of drought treatment (D0), at the end of drought stress (D17) and at the end of recovery (D22). The youngest fully expanded leaves were sampled between 8 and 9 a.m. at the end of drought stress and again at the end of recovery.

### Leaf relative water content analysis

The youngest fully expanded leaves were removed and weighed immediately for measurement of fresh weight (FW). Turgid weight (TW) was determined after leaf segments were immersed in distilled water for 6 h, and dry weight (DW) was measured after leaf segments were dried at 70°C in an oven for 24 h. Each treatment included five replications. The relative water content (RWC) was calculated as: RWC $=\frac{\text{FW}-\text{DW}}{\text{TW}-\text{DW}}\times 100\%$.

### Leaf water potential analysis

The predawn water potential of the youngest fully expanded leaves was measured using a pressure chamber (Model 3500, Soilmoisture Corp., Santa Barbara, CA, USA) between 5:30 and 6:30 a.m., based on the protocol of Chen et al. (2015). Each treatment included five replications.

### Leaf osmotic potential analysis

The youngest fully expanded leaves were used for osmotic potential measurement. Frozen leaf samples were placed into 0.5 mL tubes and allowed to thaw at room temperature. After thawing, these 0.5 mL tubes were drilled at the bottom, placed inside 1.5 mL tubes, and then centrifuged at 4000 rpm for 5 min to gather the cell sap. The osmolarity of the collected sap was determined using a dew point microvolt meter (Model 5600, Wescor, Logan, UT, USA), and the osmotic potential was calculated.

### Leaf gas exchange analysis

The photosynthetic rates, stomatal conductance, and transpiration rates of individual leaves were measured between 9:00 and 11:00 a.m. using a portable photosynthesis system (Li-6400; LI-COR Inc., Lincoln, NE, USA). The youngest fully expanded leaf was placed in the chamber at a photon flux density of 1000 μmol m^−2^ s^−1^; the flow rate through the chamber was 500 μmol s^−1^ and the leaf temperature was 28°C. The ambient CO_2_ concentration was approximately 380 μmol CO_2_ mol^−1^ air, and the vapor pressure deficit was approximately 2.0 kPa. Each treatment included five replications.

### Chlorophyll fluorescence analysis

Chlorophyll fluorescence of individual leaves was analyzed using a pulse amplitude modulated chlorophyll fluorescence system (Imaging PAM, Walz, Effeltrich, Germany) according to the method of Xu et al. (2014). The youngest fully expanded leaves were dark-adapted for 30 min before measurement. Each treatment included five replications.

### Chlorophyll and carotenoid content analysis

The pigments were extracted from frozen youngest fully expanded leaf samples (~0.2 g) using 80% acetone on a shaker at room temperature until the tissue was completely bleached. The extract was centrifuged at 5000 g for 5 min, and the supernatant was gathered for absorbance measurement at 646, 470, and 663 nm using a spectrophotometer (UV-2550, Shimadzu, Japan). The concentration of each pigment was calculated according to the method of Lichtenthaler (1987). The chlorophyll and carotenoid contents (mg g^−1^ DW) were then calculated.

### H_2_O_2_ content analysis

Hydrogen peroxide levels were determined according to the method of Yin et al. (2010). Leaf tissues (0.5 g) were homogenized in an ice bath with 5 ml 0.1% (w/v) TCA. The homogenate was centrifuged at 12,000 g for 20 min and 0.5 ml of the supernatant was added to 0.5 ml 10 mM potassium phosphate buffer (pH 7.0) and 1 ml 1 M KI. The absorption of the supernatant was read at 390 nm, and the content of H_2_O_2_ was quantified based on the standard curve.

### Lipid peroxidation analysis

Lipid peroxidation was determined through measurement of the malondialdehyde (MDA) content. Leaf material (0.5 g) was homogenized in 5 ml 0.1% (w/v) TCA solution. The homogenate was centrifuged at 12,000 g for 20 min and 0.5 ml of the supernatant was added to 1 ml 0.5% (w/v) TBA in 20% TCA. The mixture was incubated in boiling water for 30 min; the reaction was stopped by placing the reaction tubes in an ice bath. The samples were then centrifuged at 10,000 g for 5 min and the supernatant was used for an MDA assay. The MDA content was measured according to the method of Yin et al. (2010).

### Carbohydrate content analysis

Dried leaf samples were ground to a fine powder for carbohydrate analysis. Leaf powder (~0.2 g) was extracted with 6 ml of 80% (v/v) ethanol for 30 min in a water bath at 80°C under agitation and centrifuged at 5000 g for 10 min. The supernatant was then collected. The process was repeated three times. The supernatant was combined and diluted with water to 25 ml; 2 ml of this solution was taken and evaporated in a boiling water bath. The samples were dissolved in 10 ml distilled water, and the soluble sugars were measured using the anthrone reagent according to Yemm and Willis (1954).

The ethanol-insoluble residue was extracted for starch and measured using the anthrone reagent according to Clegg (1956). The pellet was re-suspended in 2 ml of water and incubated in a boiling water bath for 15 min. Two ml of 9.2 M perchloric acid was added and stirred for 15 min. The extract solution was centrifuged at 5000 g for 10 min, and the supernatant was collected. The residue was extracted again using perchloric acid, and the supernatant was combined and diluted with water to 50 ml. The starch content was then determined using the anthrone reagent. Total non-structural carbohydrates (TNC) was calculated as the sum of soluble sugars and starch.

### Nitrogen content and C/N ratio analysis

Finely ground leaf samples (~0.1 g each) were digested in H_2_SO_4_-H_2_O_2_. Nitrogen content was determined by the standard macro-Kjeldahl procedure using a Kjeltec 2300 analyzer unit (Foss Tecator AB, Hoganas, Sweden). The C/N ratio was calculated as the ratio of TNC to nitrogen content according to Chen et al. (2015).

### Growth analysis

At each time point, the shoots of 10 seedlings per treatment in each line were harvested and dried in an oven at 70°C for 72 h to determine the total dry weight (DW).

### Drought resistance, drought recovery and drought adaptability analysis

Drought resistance was estimated based on relative growth during drought stress.

$$\text{Drought resistance}=\frac{{\text{DW}}_{\text{Treatment D}17}-{\text{DW}}_{\text{Treatment D}0}}{{\text{DW}}_{\text{Control D}17}-{\text{DW}}_{\text{Control D}0}}.$$

Drought recovery was estimated based on relative growth during drought recovery.

$$\text{Drought recovery}=\frac{{\text{DW}}_{\text{Treatment D}22}-{\text{DW}}_{\text{Treatment D}17}}{{\text{DW}}_{\text{Control D}22}-{\text{DW}}_{\text{Control D}17}}.$$

Drought adaptability was estimated based on relative growth during the entire drought stress and recovery cycle.

$$\text{Drought adaptability}=\frac{{\text{DW}}_{\text{Treatment D}22}-{\text{DW}}_{\text{Treatment D}0}}{{\text{DW}}_{\text{Control D}22}-{\text{DW}}_{\text{Control D}0}}.$$

### Statistical analysis

All results are represented as means ± SE. Above-ground dry weight was measured with 10 replications, while all physiological parameters were determined in quintuplicate. Statistical analysis was performed using SPSS statistics software (Version 19.0 for Windows, SPSS, Chicago, IL, USA). Data were analyzed by analysis of variance (ANOVA) using the least significant differences (LSD) *post-hoc* test, and a *P* < 0.05 was considered significant. Principle component analysis (PCA) was performed using the relative values of all physiological traits to comprehensively evaluate the differences in plant physiological responses among the lines, and the final total score was calculated to represent physiological responses according to the method of An et al. (2013). Pearson correlations were calculated to determine the relationship among the drought-adaptive capabilities of the various lines and the relationship between their drought-adaptive capabilities and their physiological parameters or total scores of physiological responses.

## Results

### Genotypic variation of physiological response to progressive drought stress and recovery

**Figure 2:**
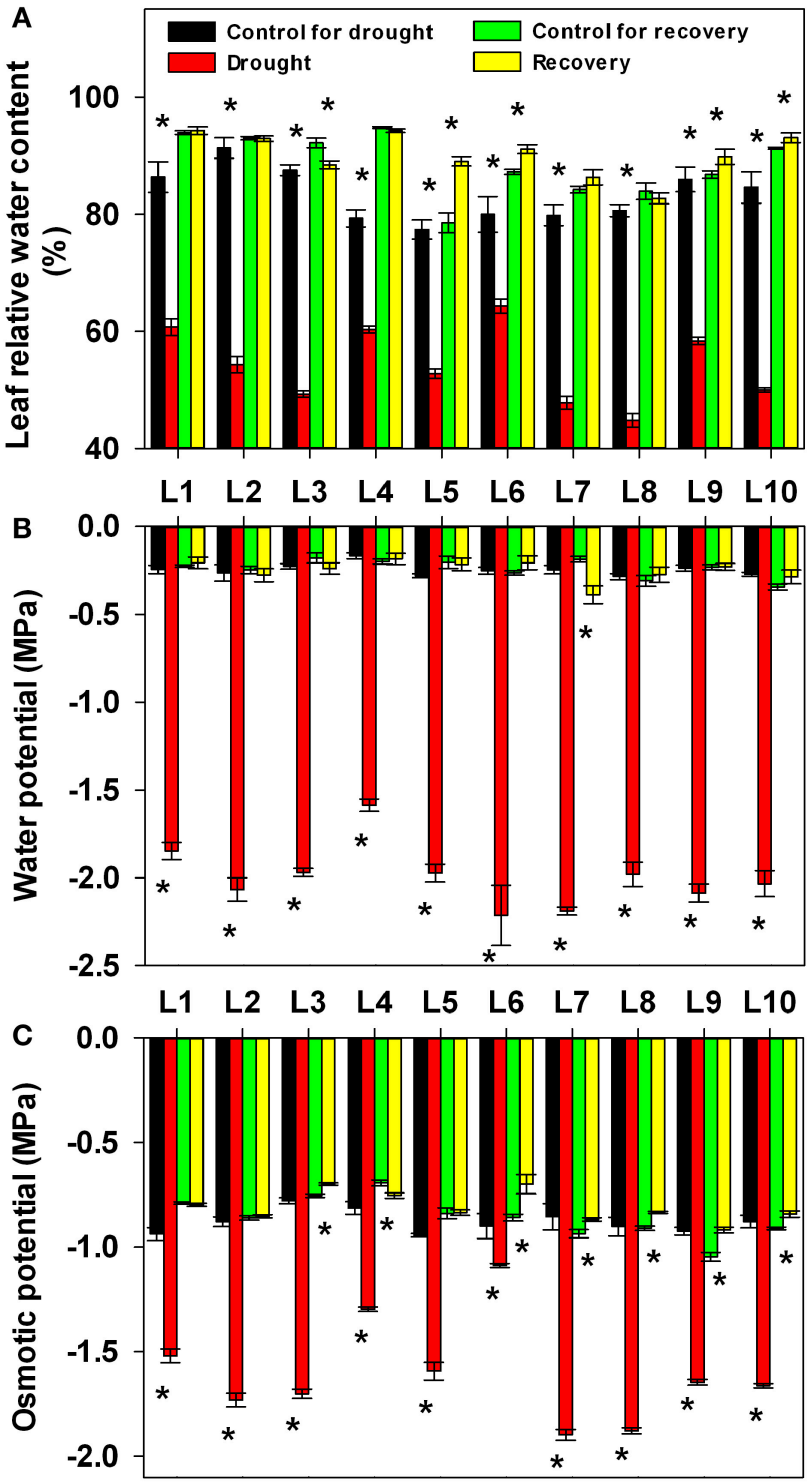
**Changes in relative water content (A), water potential (B) and osmotic potential (C) in 10 maize lines during drought stress and re-watering**. Data represent the mean ± SD (*n* = 5). Asterisks indicate statistically significant differences between treatments (^*^P ≤ 0.05).

To mimic natural drought stress in the field, a cycle of progressive drought stress and subsequent recovery was imposed by withholding and then reintroducing watering. Soil water content in all the pots in each treatment was adjusted to the same level based on daily measurements of pot weight for all maize lines (Figure 1). In order to investigate the physiological basis of genotypic variation in drought adaptation, several drought-related physiological parameters were determined. At first, we quantified the impact of progressive drought and subsequent re-watering on plant water status with detailed data on leaf RWC, leaf water potential and leaf osmotic potential from experimental plants (Figure 2). Drought stress consistently and significantly reduced leaf RWC, leaf water potential and leaf osmotic potential in comparison to controls in all maize lines. Nevertheless, the different lines responded differently to drought stress. Among the lines we examined, L6 had the highest leaf water content at 64.3%, while L8 had the lowest at 44.8%. L4 had the highest leaf water potential at −1.59 MPa, while L6 had the lowest at −2.21 MPa. The osmotic potential ranged from −1.90 MPa in L7 to −1.08 MPa in L6. After re-watering, the leaf RWC, leaf water potential and leaf osmotic potential returned essentially to control levels in all lines. Yet both treatment history and line had a significant effect on the final values of these parameters at the end of the recovery period. The interaction between treatment history and line also significantly affected RWC and leaf osmotic potential, though this had no significant effect on leaf water potential (Supplementary Table 1).

**Figure 3:**
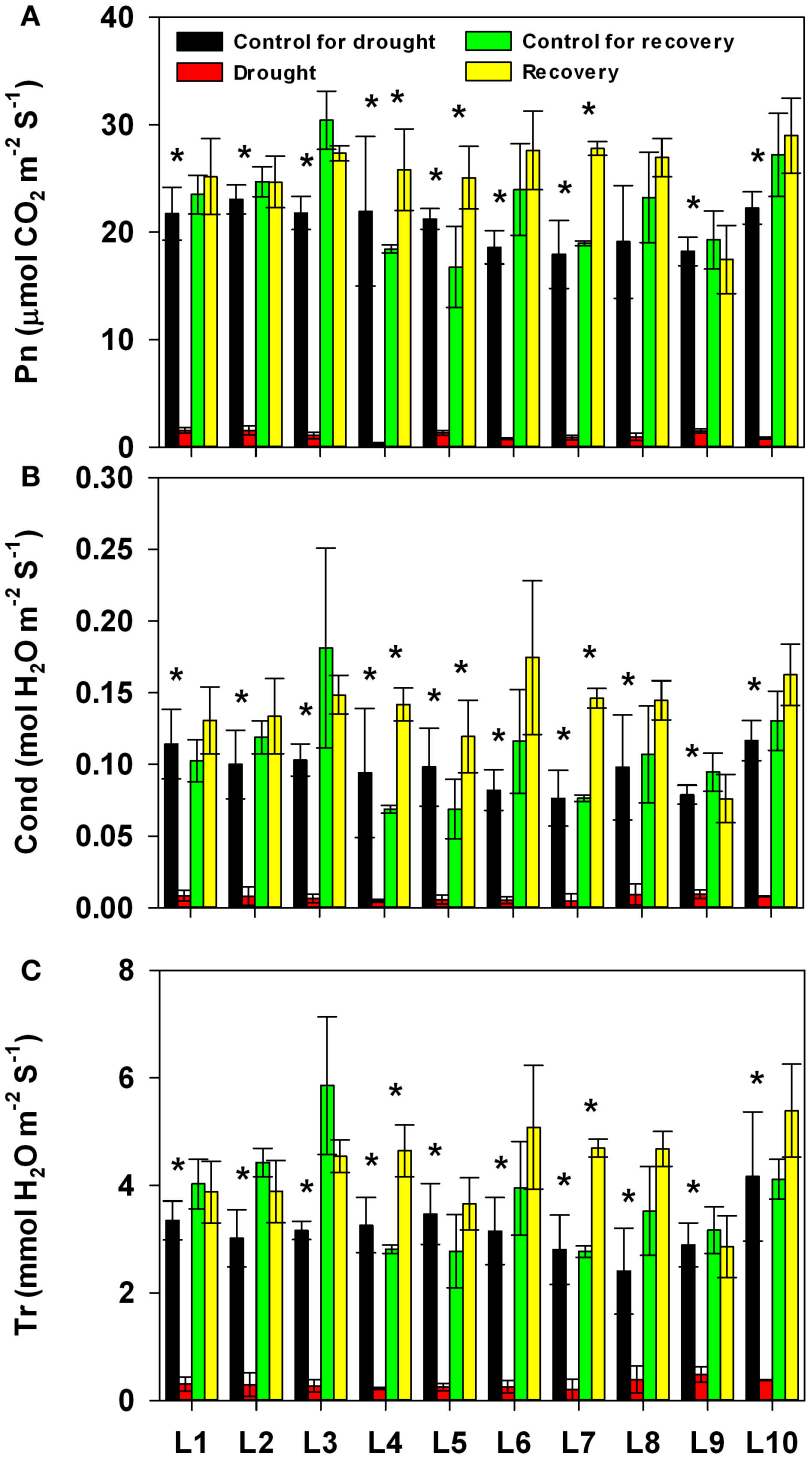
**Changes in photosynthesis rate (Pn, A), stomatal conductance (Cond, B), and transpiration rate (Tr, C) in 10 maize lines during drought stress and re-watering**. Data represent the mean ± SD (*n* = 5). Asterisks indicate statistically significant differences between treatments (^*^P ≤ 0.05).

We next addressed the impact of progressive drought and subsequent re-watering on gas exchange parameters (Figure 3 and Supplementary Table 1). Photosynthetic rate, stomatal conductance and transpiration rate were almost completely suppressed by the prolonged drought stress; line had no significant effect on the severity of this suppression. After re-watering, the gas exchange parameters recovered rapidly in all lines, though both treatment history and line, as well as the interaction between the two, had a significant effect on the recovery of all parameters; the sole exception to this was that the interaction between treatment history and line did not affect Cond. It is worth noting that, after re-watering, the photosynthetic rate was significantly higher in drought-treated plants of the L4, L5, and L7 lines than in control plants of the same lines. Correspondingly, these lines also had higher stomatal conductance.

**Figure 4:**
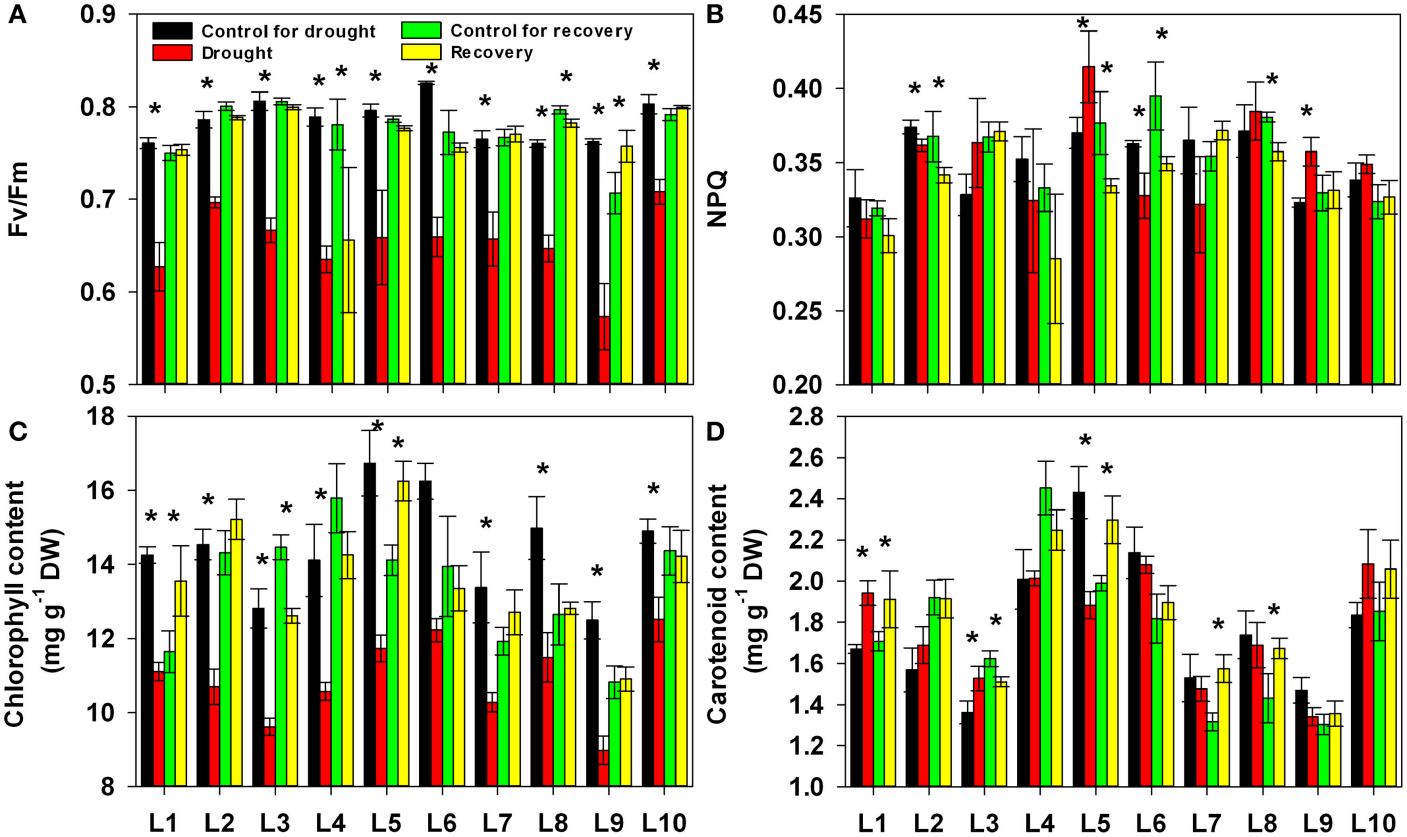
**Changes in maximum efficiency of PSII photochemistry (Fv/Fm, A), non-photochemical quenching (NPQ, B), chlorophyll content (C) and carotenoid content (D) in 10 maize lines during drought stress and re-watering**. Data represent the mean ± SD (*n* = 5). Asterisks indicate statistically significant differences between treatments (^*^P ≤ 0.05).

Following our analysis of the gas exchange parameters, we investigated the changes in chlorophyll fluorescence and contents of photosynthetic pigment, chlorophyll and carotenoid under progressive drought and subsequent re-watering. As shown in Figure 4A, drought stress consistently and significantly reduced the maximum efficiency of PSII photochemistry (Fv/Fm), though this effect varied in its severity among the different lines (Supplementary Table 1). After re-watering, the Fv/Fm recovered to control levels in all lines. There were no obvious upward or downward trends in non-photochemical quenching (NPQ) values under either drought stress or re-watering conditions. Drought stress had no significant effect on the NPQ (Figure 4B). The chlorophyll content was consistently and significantly reduced by drought stress, and the degree of this reduction was significantly affected by line as well as by the interaction between line and treatment history. After re-watering, the chlorophyll content recovered to control levels in all lines (Figure 4C). The carotenoid content differed among lines, but drought stress had no significant effect on it (Figure 4D).

**Figure 5:**
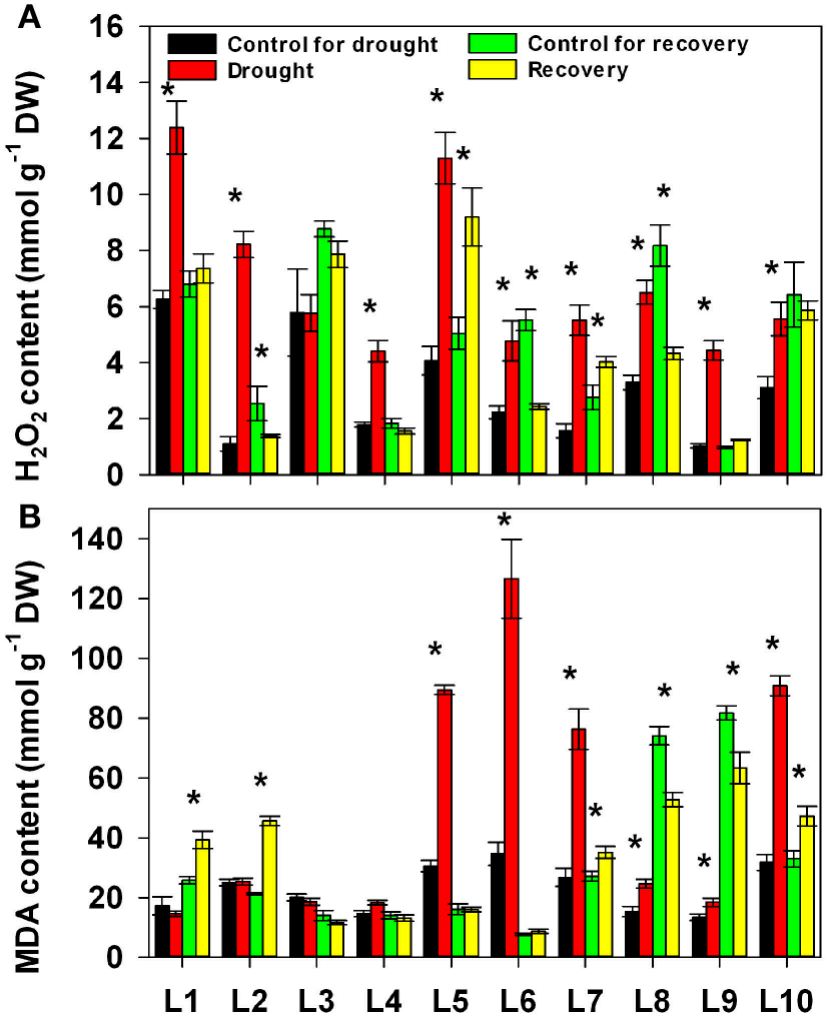
**Changes in H_2_O_2_ content (A) and MDA content (B) in 10 maize lines during drought stress and re-watering**. Data represent the mean ± SD (*n* = 5). Asterisks indicate statistically significant differences between treatments (^*^P ≤ 0.05).

We next addressed the impact of progressive drought and subsequent re-watering on hydrogen peroxide (H_2_O_2_) and lipid peroxidation (Figure 5). Both drought and re-watering had a significant effect on H_2_O_2_ accumulation: it significantly increased under drought stress and decreased after re-watering in most lines. Both the increase and the decrease were significantly affected by line and by the interaction between treatment and line (Supplementary Table 1). As for MDA accumulation, the lines responded differently to both drought stress and re-watering treatment. There were no obvious change in MDA levels in L1~4 under drought stress, whereas drought markedly stimulated MDA accumulation in L5, L6, L7, and L10. After re-watering, MDA decreased back to normal levels in L5, L6, L7, and L10, while it notably increased in L1 and L2.

**Figure 6:**
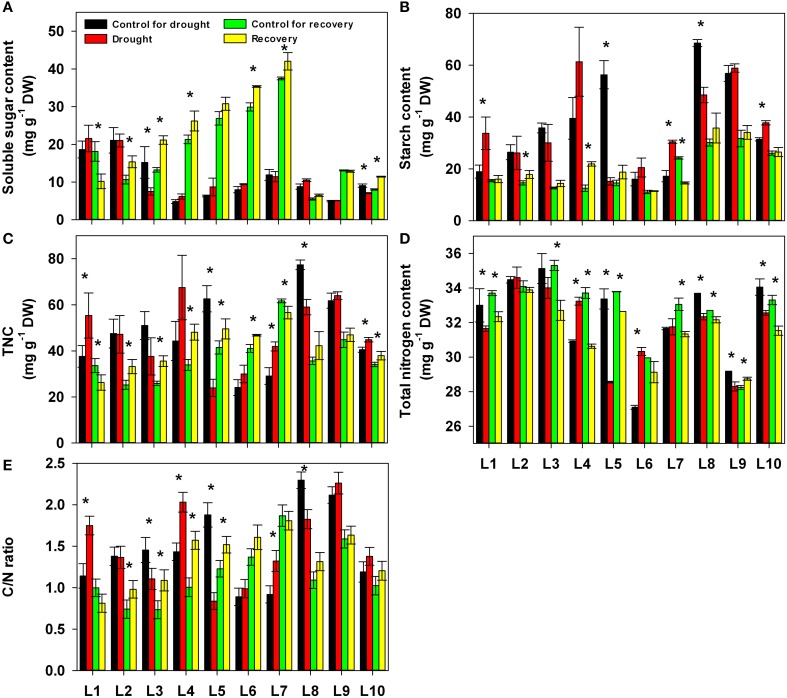
**Changes in carbohydrates, nitrogen compounds and the C/N ratio in 10 maize lines during drought stress and re-watering**. **(A)** Soluble sugars. **(B)** Starch. **(C)** Total non-structural sugars (TNC). **(D)** Total nitrogen content. **(E)** C/N ratio. Data represent the mean ± SD (*n* = 5). Asterisks indicate statistically significant differences between treatments (^*^P ≤ 0.05).

We also quantified the changes in carbohydrates and nitrogen content under drought stress and after re-watering (Figure 6). Drought stress was not associated with any obvious upward or downward trend in any of these parameters. Re-watering, in most lines, significantly increased the content of soluble sugar and the total non-structure carbohydrates (TNC) as well as the C/N ratio and decreased the total nitrogen content compared with control plants in the same lines. Neither drought nor re-watering significantly affected the starch content. For all these parameters, the different lines exhibited different responses to both drought stress and re-watering treatment (Supplementary Table 1).

### Overall assessment of the physiological responses

**Figure 7:**
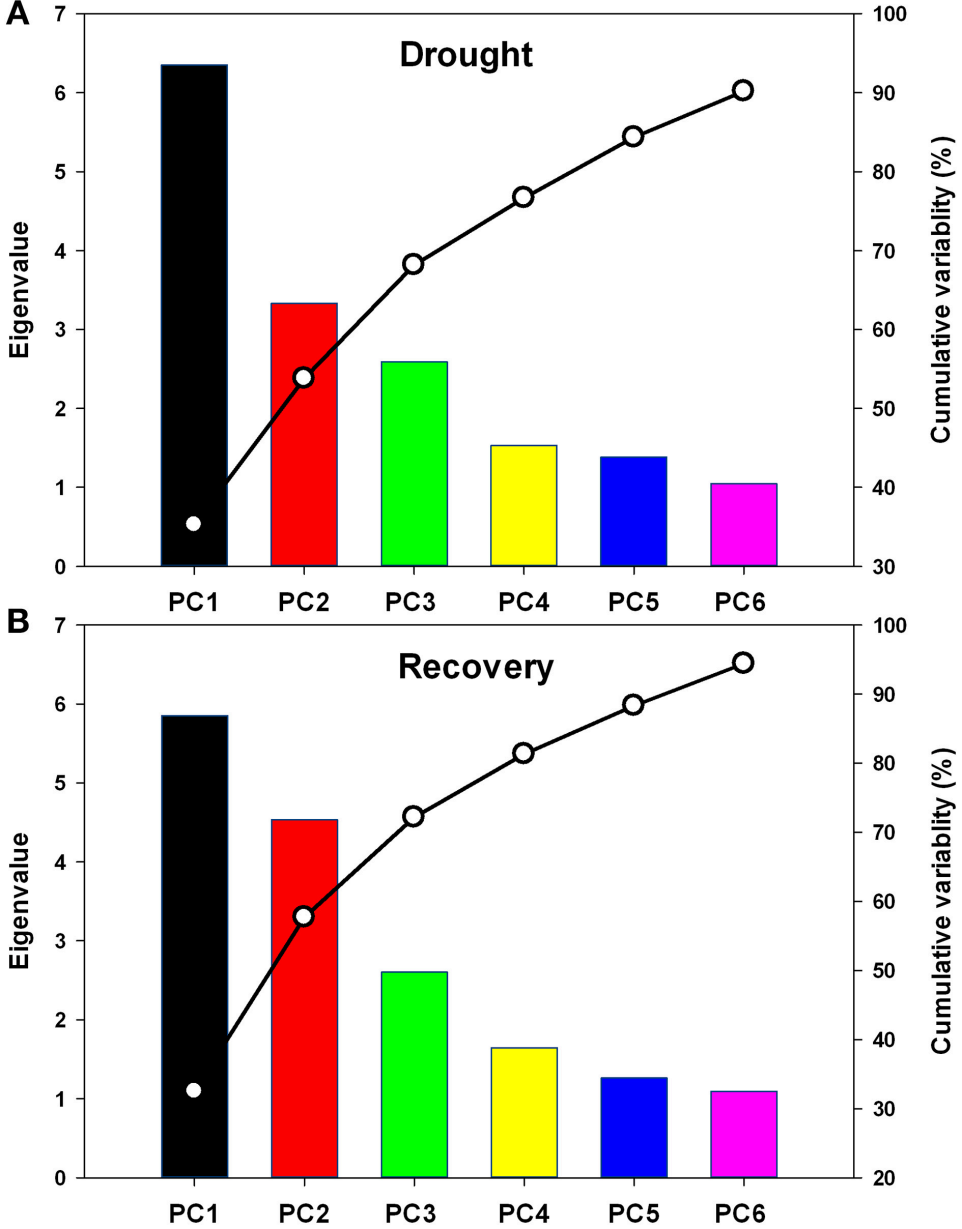
**Scree plot of variance explained by each factor of the principal component during drought stress (A) and re-watering (B)**. PC1-PC6: the first to the sixth principal component.

Further, complete data sets, one showing the relative physiological changes under drought stress and another showing these changes under recovery, were subjected to principal component analysis. Six principal components (PC1-6) were extracted, and together these dimensions explained over 90 and 94% of the total variability under drought stress and recovery, respectively (Figure 7). The data dimensions were therefore reduced from eighteen to six for further data processing.

**Figure 8:**
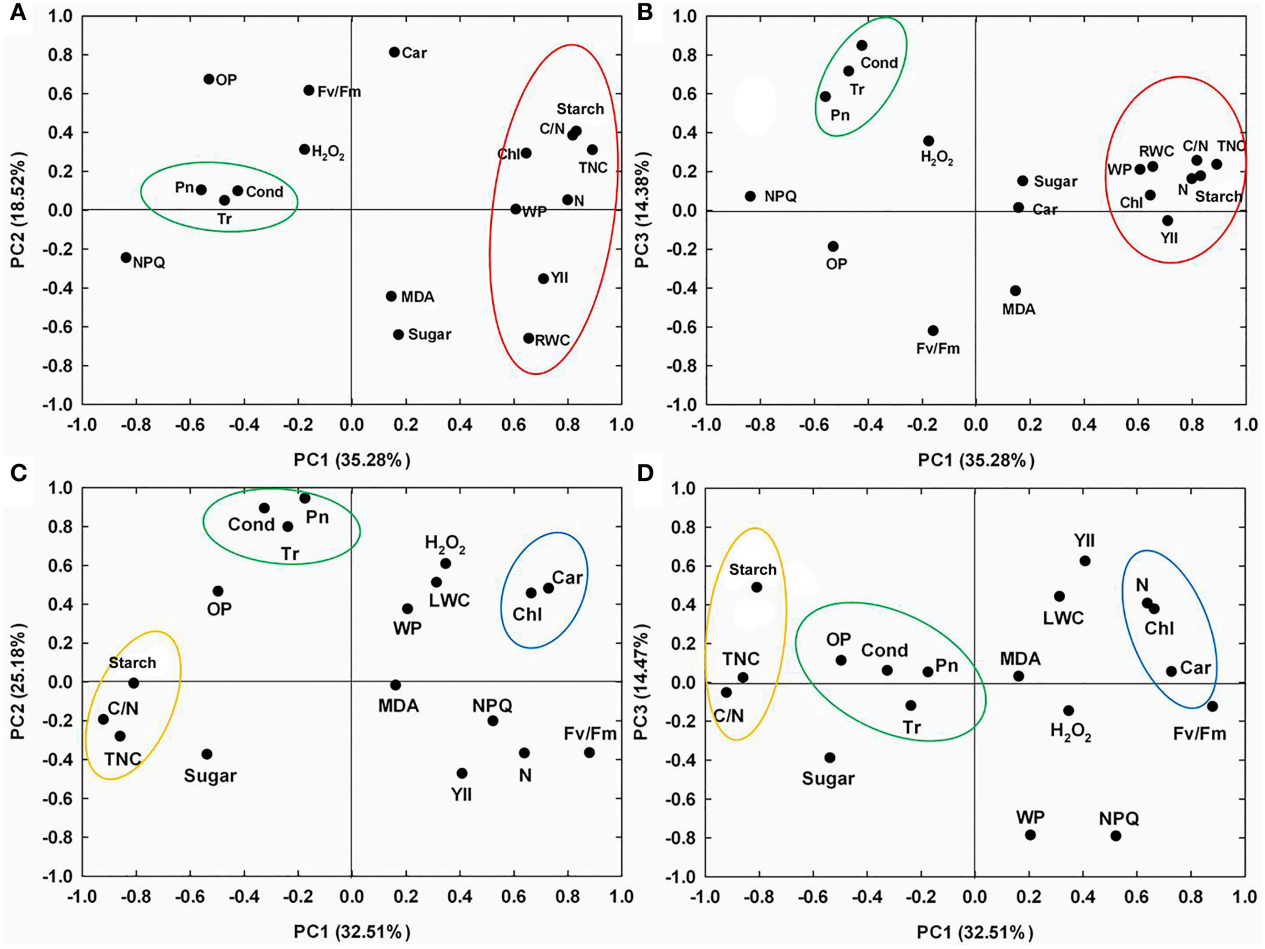
**Principal component analysis (PCA) for physiological responses in 10 maize lines**. PC1-PC2 and PC1-PC3 variables loading plots during drought stress **(A,B)** and re-watering **(C,D)**. PC1-PC3: the first, second, and third principal component.

As shown in Figures 8A,B, depicting the responses to drought stress, PC1 was heavily associated with total non-structural carbohydrates content, starch content, C/N ratio, nitrogen content, effective PSII quantum yield, leaf RWC, chlorophyll content, water potential and non-photochemical quenching coefficient (negatively); PC2 gave a high weighting to carotenoid content, osmotic potential and Fv/Fm; and PC3 was associated with gas exchange parameters. As shown in Figures 8C,D, depicting the responses to recovery, PC1 was heavily associated with Fv/Fm, pigment content and nitrogen content (positively), and total non-structural carbohydrates content, starch content, and C/N ratio (negatively); PC2 gave a high weighting to gas exchange parameters; and PC3 was negatively associated with non-photochemical quenching coefficient and water potential.

The principal components reflected different aspects of physiological responses. To display the genotypic variation of the physiological responses more intuitively, scores representing total responses to drought and recovery were calculated for each line (Table 1). Under drought, L1 showed the strongest response, while L5 showed the weakest response. Under recovery, L4 showed the strongest response, while L7 showed the weakest response.

**Table 1:** **Total score of physiological responses of different lines**.

**Line**	**L1**	**L2**	**L3**	**L4**	**L5**	**L6**	**L7**	**L8**	**L9**	**L10**
Drought response	1.28	0.14	−0.97	1.02	−2.20	0.98	0.82	−0.80	−0.35	0.08
Recovery response	0.38	−0.62	−1.48	1.47	0.06	1.16	−1.73	0.86	0.02	−0.12

### Genotypic variation of growth response to progressive drought stress and recovery

**Figure 9:**
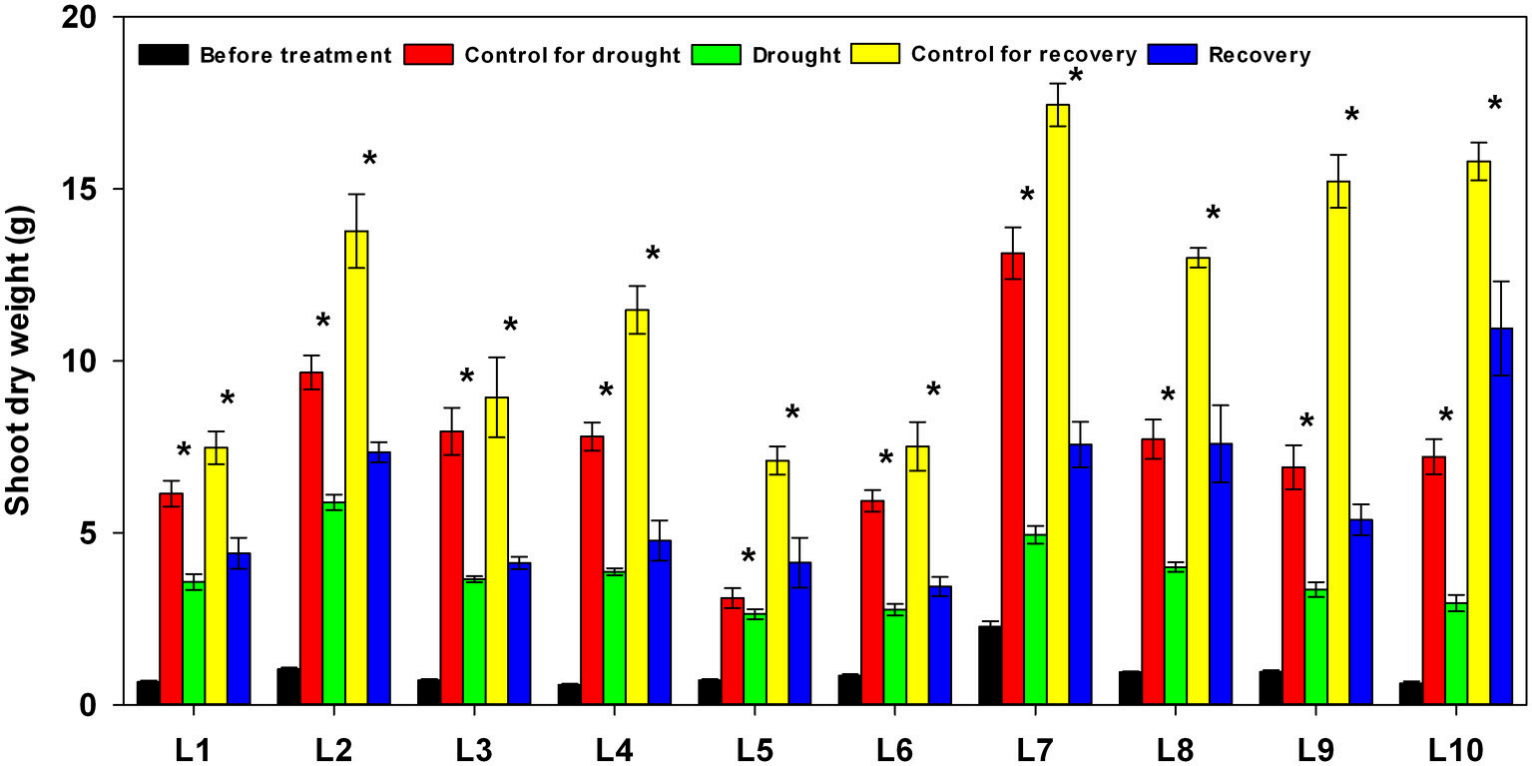
**Changes in shoot dry weight in 10 maize lines during drought stress and re-watering**. Asterisks indicate statistically significant differences between control and treatment (^*^P ≤ 0.05).

The tissues above ground were harvested at the start of treatment, at the end of drought stress, and again at the end of recovery. As shown in Figure 9, during the 17 days of drought stress and the 5 days of re-watering, control plants in all maize lines showed a significant increase in total shoot dry weight. Plants subjected to drought stress, on the other hand, showed a significant decrease in above-ground biomass accumulation in all lines, yet the severity of the adverse effects of drought stress on growth varied among the lines: the biomass seen in drought-stressed plants ranged from 37.6% in L7 to 85.0% in L5 compared to well-watered plants of the same lines. After re-watering, the above-ground biomass ranged from 35.3% in L9 to 69.3% in L10 compared to well-watered plants.

### Genotypic variation of drought resistance, recovery and adaptability

**Figure 10:**
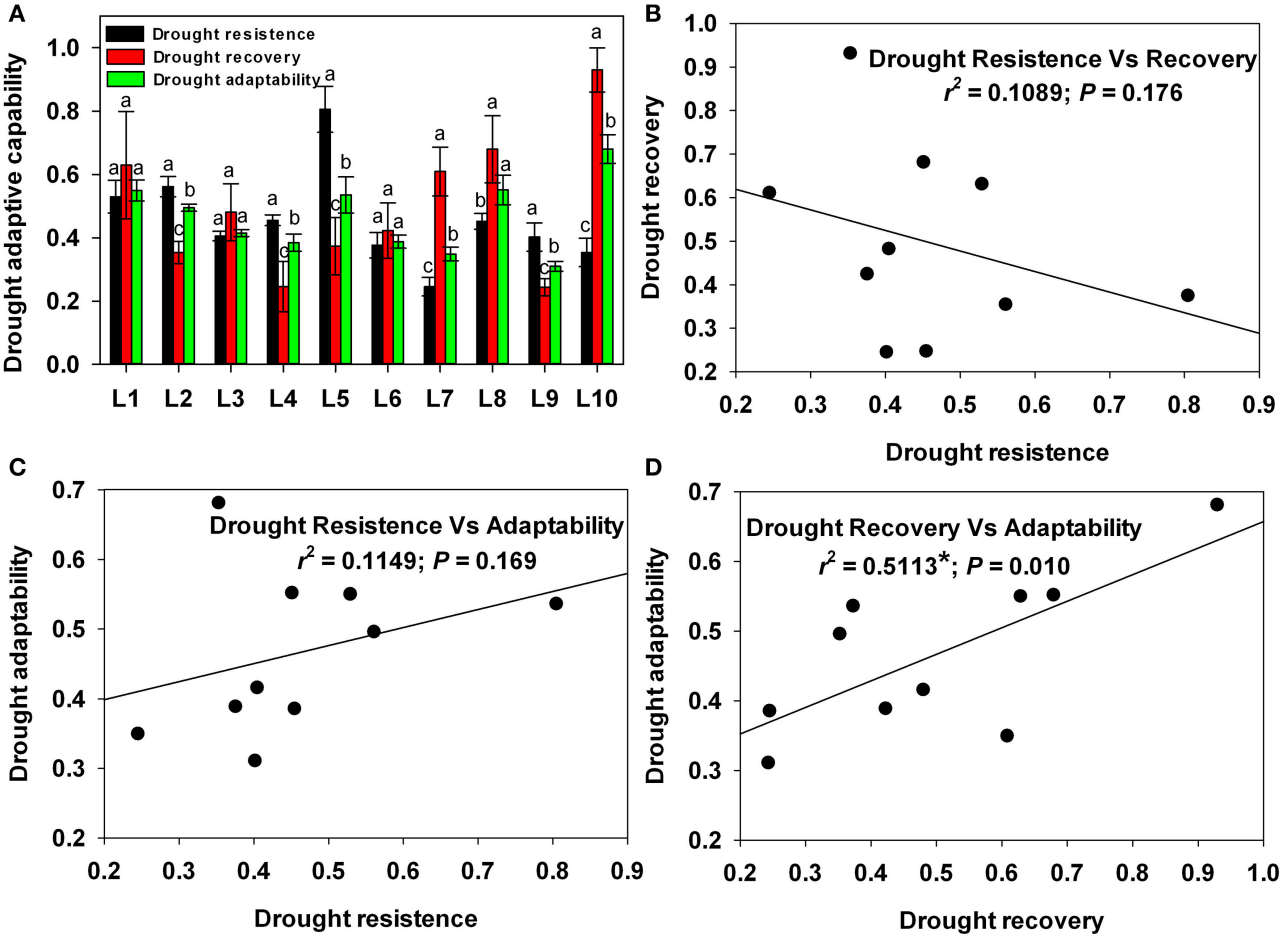
**Drought-adaptive capabilities and correlations between drought-adaptive capabilities. (A)** Drought-adaptive capabilities. **(B)** Correlation between drought resistance and drought recovery. **(C)** Correlation between drought resistance and drought adaptability. **(D)** Correlation between drought recovery and drought adaptability. Different letters and asterisks indicate statistically significant differences between drought-adaptive capabilities (^*^P ≤ 0.05).

To enable a comparative analysis of the roles of drought resistance and drought recovery in drought adaptation, each line's drought resistance, recovery and adaptability were estimated based on relative growth during drought stress, recovery and the entire cycle, respectively. As shown in Figure 10A, drought resistance, recovery and adaptability all showed substantial variation among the lines. L5 showed the strongest drought resistance at up to 0.806, while L7 showed the weakest at 0.246. L10, meanwhile, showed the strongest drought recovery and adaptability, while L9 showed the weakest.

Next, in order to analyze the relationship between drought resistance and recovery, drought recovery was regressed against drought resistance (Figure 10B). There was little correlation (*r*^2^ = 0.11) between resistance and recovery. This result indicates that drought recovery was independent of drought resistance.

Strong drought adaptability can result from either strong resistance or strong recovery. To determine which factor contributes more to drought adaptability, drought adaptability was regressed against drought resistance (Figure 10C) and against drought recovery (Figure 10D). Drought adaptability had little correlation (*r*^2^ = 0.11) with resistance but was highly correlated with recovery (*r*^2^ = 0.51^**^). This result indicates that drought recovery played a more important role than resistance in drought adaptation among the maize lines we examined.

### Correlation analysis between physiological traits and drought-adaptive capabilities

**Figure 11:**
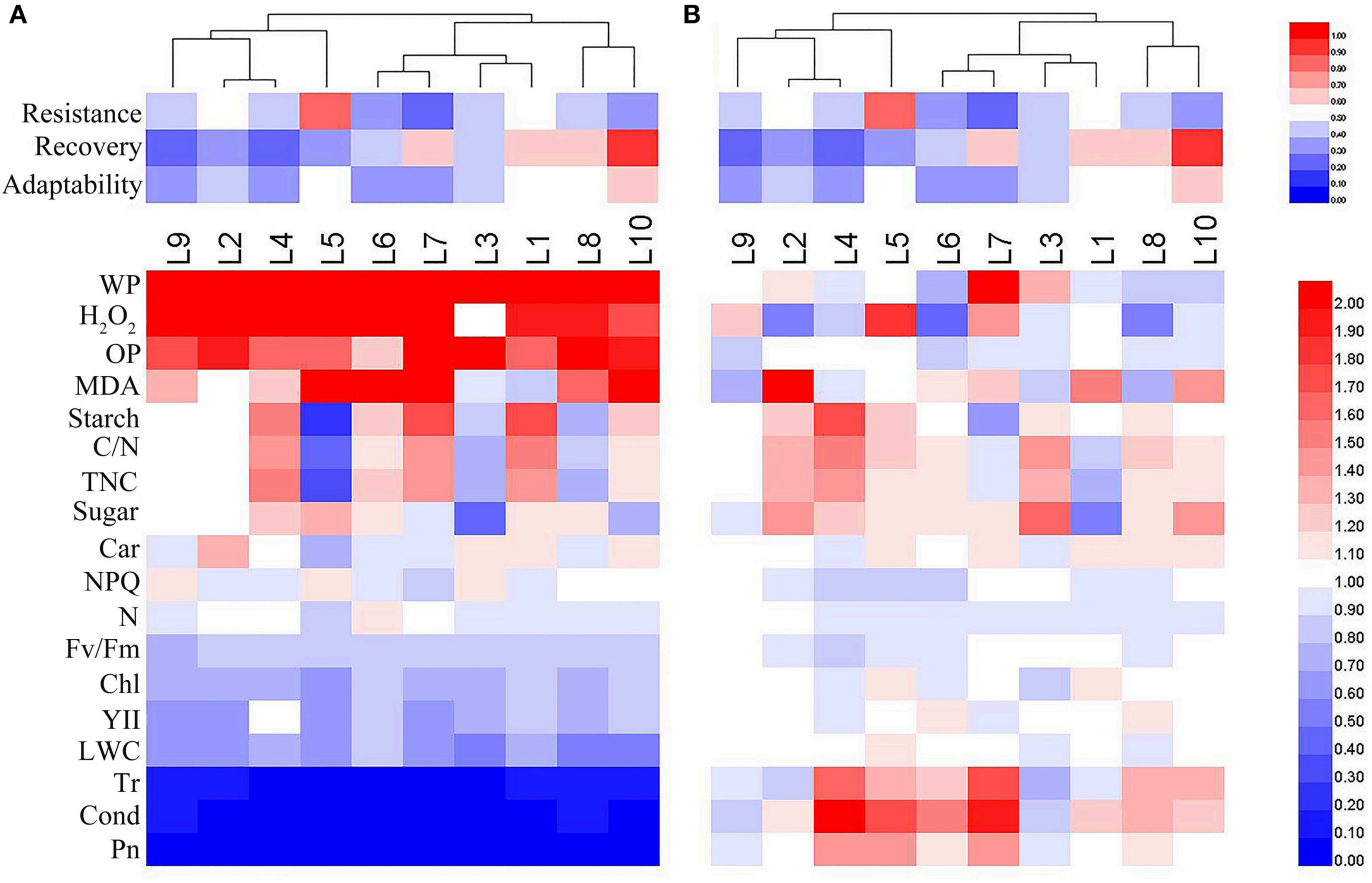
**Overall assessments of physiological responses in 10 maize lines during drought stress (A) and re-watering (B)**. The relative values of physiological parameters for the heat map analysis. Scale: for the drought adaptation capabilities, from brightest blue equal lowest to brightest red equal highest capabilities; for physiological parameters, from brightest blue equals most decreased to brightest red equals most increased.

The heat map in Figure 11 summarizes overall assessments of the physiological responses to drought stress and recovery in each of the maize lines sorting by the drought adaptation performance. The heat map clearly reveals considerable variation among the lines in their physiological responses to progressive drought stress and recovery. Under drought stress, the relative values of water potential, H_2_O_2_ content and osmotic potential increased in all lines, while the Fv/Fm, chlorophyll content, effective PSII quantum yield, leaf RWC, transpiration rate, stomatal conductance and photosynthetic rate dropped in all lines; other responses varied among the lines (Figure 11A). After recovery, most of the measured traits recovered to control levels, but there was still considerable variation in physiological parameters among the lines (Figure 11B).

To define the roles of physiological responses in plant drought adaption, correlation analysis between drought-adaptive capabilities and total scores in physiological responses was conducted (Table 2). The only significant correlation found was a negative correlation between total physiological response score under drought stress and drought resistance (*r* = −0.58, *P* < 0.05). Then, to characterize the physiological traits possibly related to drought-adaptive capabilities in greater detail, the correlations between relative values of physiological changes and drought-adaptive capability were analyzed (Table 2). Under drought stress, the water potential, chlorophyll content, starch content, total non-structural carbohydrates content, nitrogen content and C/N ratio were negatively associated with drought resistance; Fv/Fm and chlorophyll content were positively associated with drought recovery; and water potential was heavily and negatively associated with drought adaptability, while Fv/Fm and chlorophyll content were positively associated with drought adaptability. After recovery, leaf RWC was positively associated with drought resistance, while non-photochemical quenching coefficient was negatively associated with drought resistance, and a significant positive correlation between carotenoid content and drought recovery was found.

**Table 2:** Correlations (*r*) between drought-adaptive capabilities and physiological responses ^*^P ≤ 0.05, ^**^P ≤ 0.01, ^***^P ≤ 0.001.

**Trait**	**Drought**			**Recovery**		
	**Resistance**	**Recovery**	**Adaptability**	**Resistance**	**Recovery**	**Adaptability**
**Total score**	−0.567^*^	0.083	−0.214	0.237	−0.193	0.119
LWC	0.144	−0.494	−0.345	0.559^*^	−0.185	0.070
**WP**	−0.554^*^	−0.509	−0.827^**^	−0.358	0.034	−0.414
OP	−0.249	0.328	0.072	0.464	−0.197	0.195
Pn	0.304	0.137	−0.044	0.220	−0.065	−0.047
Cond	−0.116	−0.109	−0.149	0.070	−0.060	−0.072
Tri	−0.12	0	−0.049	−0.222	0.145	−0.059
**Fv/Fm**	0.001	0.626^*^	0.65^*^	−0.112	0.265	0.051
NPQ	0.442	−0.06	0.211	−0.594^*^	0.460	−0.066
YII	−0.022	−0.066	0.085	0.284	0.084	0.262
**Chl**	−0.554^*^	0.874^***^	0.561^*^	0.522	0.145	0.341
Car	−0.181	0.24	0.295	0.040	0.558^*^	0.358
H2O2	0.178	−0.42	−0.192	0.328	−0.099	−0.093
MDA	−0.122	0.256	0.071	0.094	0.163	0.327
Sugar	0.522	−0.344	−0.006	−0.140	0.025	0.060
**Starch**	−0.614^*^	0.161	−0.245	0.492	−0.499	0.048
**TNC**	−0.589^*^	0.054	−0.278	0.197	−0.411	−0.085
**N**	−0.565^*^	−0.242	−0.48	0.163	−0.170	−0.064
**C:N**	−0.573^*^	0.142	−0.199	0.147	−0.363	−0.082

## Discussion

In practice, non-irrigated crops in temperate climates and irrigated crops under arid climates are subjected to continuous cycles of water stress and re-watering (Perrone et al., 2012). Recently, increasing importance has been attached to plants' capacity for drought recovery, particularly in crops (Chaves et al., 2009; Luo, 2010; Perrone et al., 2012; Vanková et al., 2012; Fang and Xiong, 2015). In the present study, the roles of drought resistance and drought recovery in drought adaptation were comparatively analyzed. Among the four maize lines with the highest drought adaptability (L1, L5, L8, L10), L5 possessed the highest drought resistance, while L8 and L10 possessed the highest drought recovery, and L1 possessed both high drought resistance and high recovery (Figure 10A). Further correction analysis revealed that drought adaptability is closely related to drought recovery, but not to drought resistance (Figures 10C,D). These results confirm that both drought resistance and drought recovery are key determinants of plant drought adaptation, and suggest that drought recovery may play the more important role in drought adaptation.

The majority of the maize lines assessed in the present study exhibited similar patterns in their physiological responses to drought stress and recovery. Consistent with the results reported in previous studies, drought induced decreases in leaf water content, water potential, osmotic potential, gas exchange parameters, chlorophyll content, Fv/Fm and nitrogen content, and increased H_2_O_2_ accumulation and lipid peroxidation (Figures 2–6; Mahouachi et al., 2006; Efeoglu et al., 2009; Flexas et al., 2009; Posch and Bennett, 2009; Bibi et al., 2010; Vassileva et al., 2011; Jiang et al., 2012; Ebrahimiyan et al., 2013; Li-Marchetti et al., 2015; Ying et al., 2015). After recovery, most of these physiological parameters rapidly returned to normal levels (Figures 2–6; Wang and Huang, 2004; Efeoglu et al., 2009; Flexas et al., 2009; Chai et al., 2010; Puangbut et al., 2010; Vassileva et al., 2011; Jiang et al., 2012; Domenghini et al., 2013). Yet although the various lines tended to exhibit similar patterns of physiological change, the amplitudes of these changes varied among the lines. Most traits were significantly affected by treatment, line, and the interaction between treatment and line (Supplementary Table 1). Further correlation analysis between physiological changes and drought-adaptive capabilities indicated that the ability to maintain a higher water potential and lower chlorophyll content, starch content, total non-structure carbohydrates, nitrogen content and C/N ratio under drought stress contributes to drought resistance; whereas the ability to maintain a higher chlorophyll content and Fv/Fm under drought stress contributes to drought recovery; and the ability to maintain a higher water potential, chlorophyll content and Fv/Fm under drought stress contributes to drought adaptability (Table 2). Furthermore, principal component analysis showed that L5, the line with the highest drought resistance, also had the weakest total score for physiological responses under drought stress (Table 1). Correspondingly, drought resistance was negatively related to total physiological response (Table 2). These results revealed that the physiological bases of drought resistance and drought recovery are definitely different, and that reducing the damage associated with drought stress on plant photosynthetic systems contributes to rapid recovery after re-watering.

Maintaining well water status is crucial to optimal physiological functioning and growth. Some studies have suggested that high RWC is closely related to drought resistance (Altinkut et al., 2001; Keles and Oncel, 2004). In the present study, however, RWC was not closely related to drought resistance, though leaf water potential, a key indicator of the degree of cell and tissue hydration, was (Table 2). This result suggested that leaf water potential may serve as an indicator of plant water status, and that a plant's ability to maintain adequate water status improves drought adaptability by enhancing drought resistance but not drought recovery.

Osmotic adjustment has been considered an important part of drought tolerance, and the indicators of drought tolerance used to date have mainly consisted of physiological parameters related to osmotic adjustment (such as osmotic potential, soluble sugar, and proline content; Wei et al., 2009; Fang and Xiong, 2015). In the present study, however, neither osmotic potential nor soluble sugar was significantly related to drought resistance or recovery (Table 2). Several studies have suggested a positive correlation between photosynthesis and osmoregulation (Shangguan et al., 1999; Hura et al., 2007). In this study, photosynthesis was strongly inhibited after prolonged soil drought but rapidly recovered to normal levels after re-watering in all lines (Figure 3). There was no significant correlation between photosynthesis and drought resistance or recovery (Table 2). This may be the reason why the osmotic potential was not related to drought resistance and recovery in this study.

Chlorophyll, a photosynthetic pigment, is involved in light absorption and plays an important role in plant photosynthesis. As drought stress can accelerate chlorophyll decomposition, chlorophyll content is one of the most frequently used metrics for the severity of drought stress (Efeoglu et al., 2009; Ying et al., 2015). As expected, we found that the chlorophyll content in all lines significantly decreased under drought stress (Figure 4C). Yet we also found a significant negative correlation between chlorophyll content and drought resistance and a significant positive correlation between chlorophyll content and drought recovery (Table 2). Maintaining lower chlorophyll content under serious drought stress may help plants reduce photo-oxidative damage, which occurs when photosynthesis is inhibited and light excitation energy is in excess (Aranjuelo et al., 2011). The excessive excitation energy absorbed by photosynthetic pigment in photosystem II will lead to an impairment of photosynthetic function, progressing to an accumulation of reactive oxygen species (ROS) and resulting in oxidative stress (Pintó-Marijuan and Munné-Bosch, 2014). Maintaining higher chlorophyll content contributes to the rapid recovery of photosynthesis. Similarly, drought resistance had no correlation with Fv/Fm, while drought recovery was positively associated with Fv/Fm (Table 2). Taken together, our results indicate that the ability to preserve the stability of the photosynthetic system during drought and re-watering enhances drought adaptability because it improves drought recovery, though it does not improve drought resistance.

In selecting drought-adaptive genotypes for breeding, it is helpful to have physiologically trustworthy indicators of drought-adaptive capabilities (Hura et al., 2007). In the present study, the correlation analysis between relative values of physiological changes and drought-adaptive capabilities indicated that leaf water potential, chlorophyll content and Fv/Fm under drought stress could be used as reliable reference indicators in the selection of drought-adaptive genotypes. All three indicators were related to drought adaptability; more specifically, leaf water potential and chlorophyll content were related to drought resistance while chlorophyll content and Fv/Fm were related to drought recovery (Table 2).

The present study suggests that drought recovery may play a more important role than drought resistance in drought adaptation. Reduced drought-associated damage to plant photosynthetic systems is the basis of rapid recovery after re-watering. Recently, increasing importance has been attached to drought recovery in crops, particularly in light of global climatic change (Chaves et al., 2009; Luo, 2010; Perrone et al., 2012; Vanková et al., 2012; Fang and Xiong, 2015). Fang and Xiong (2015) regard drought recovery as one of the major components of drought resistance, along with drought avoidance, drought tolerance and drought escape. In the present study, drought adaptability was closely related to drought recovery, but not to drought resistance. Further, it is obvious that the physiological bases of drought resistance and drought recovery are different. Therefore, although both drought resistance and recovery are key determinants of plant drought adaptation, drought recovery may play the more important role.

In the present study, the drought adaptive capabilities were estimated based on the seedling relative growth. Yield is determined by growth and developmental processes and plant growth was considered as a measure of drought adaptive capacity (Blum, 1979; Dolferus, 2014). As the seedlings behavior in growth associate with yield under drought, it could partly reflect the potential of drought adaptability (Liu et al., 2013; Ramegowda et al., 2014; Yang et al., 2014). And drought recovery may also play an important role in the whole growth period. It has been reported that seedlings behavior does not absolutely reflect general field stress adaptability (Katerji et al., 2012). At the flowering and grain filling stages, when the crops are susceptible to water deficit stress and the dry matter accumulation are not the yield-limited factor, the role of drought recovery may be limited. Thus, the role of drought recovery should be further confirmed during whole growth period along with yield under field condition. In addition, it is worth noting that the number of maize lines used in this study may not have been large enough to permit us to see all of the extant genotypic variation of drought adaptation. And for practical reasons we were not able to perform dynamic monitoring of the numerous physiological responses during the recovery process, and this prevented us from comprehensively investigating the physiological bases of drought recovery. Therefore, our findings on the physiological bases and potential indicators of drought adaptation are limited and need to be confirmed in future studies. More detailed investigations sampling more variables at more time points along with final grain yields will provide us with a better understanding of drought-adaptive mechanisms.

**Figure 12:**
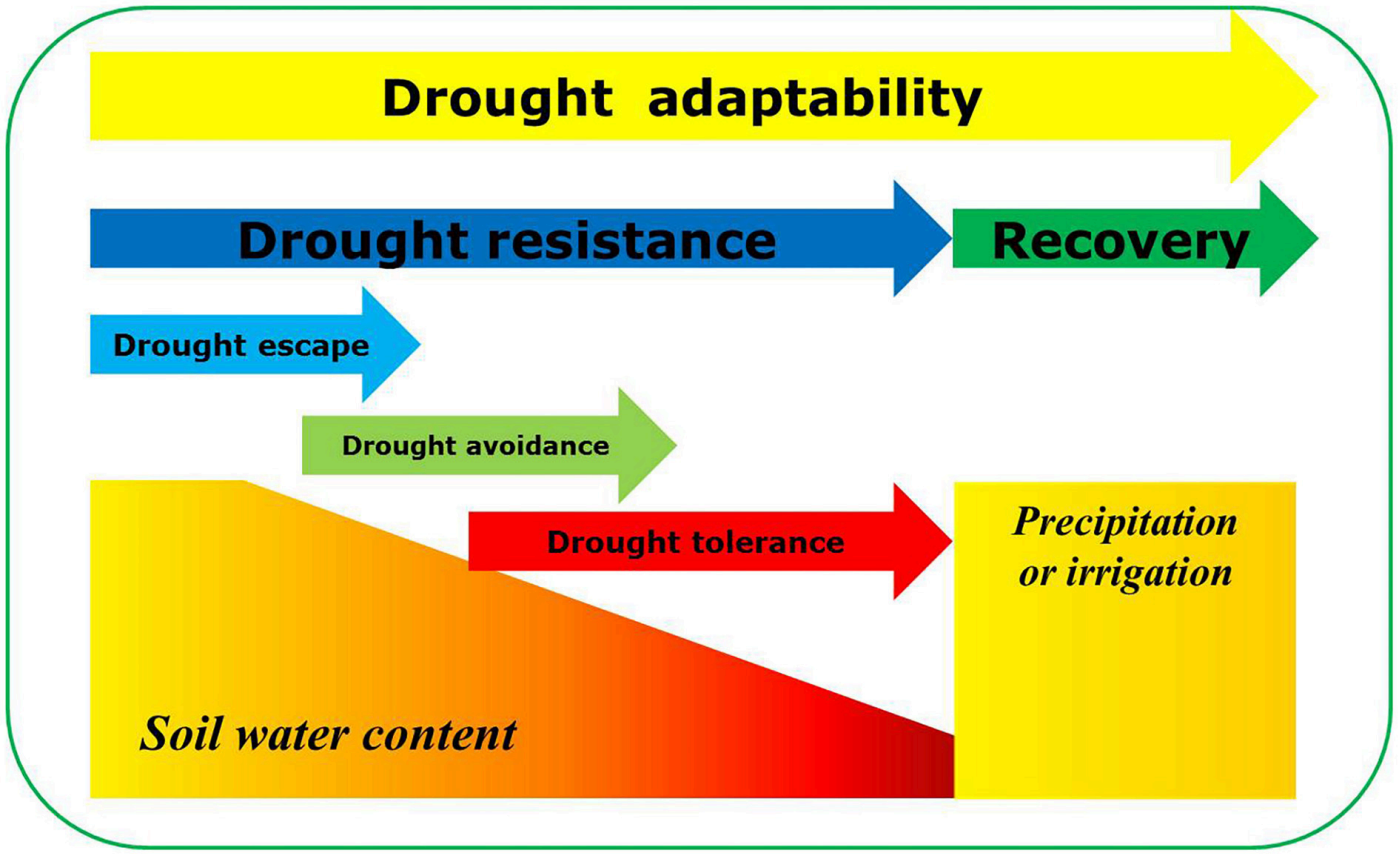
**Diagram depicting drought-adaptive capabilities during drought and re-watering cycle**. Drought adaptability was defined as comprehensive capacity for adaptation to the drought stress and re-watering cycle. Both drought resistance and recovery are key determinants of plant drought adaptation. Drought resistance involves three major mechanisms: drought escape, drought avoidance and drought tolerance.

In conclusion, the present study suggests that leaf water potential, chlorophyll content and Fv/Fm under drought stress could be used as reference indicators in the selection of drought-adaptive genotypes as leaf water potential and chlorophyll content were related to drought resistance while chlorophyll content and Fv/Fm could indicating drought recovery. Although both drought resistance and recovery are key determinants of seedling plant drought adaptation, drought recovery may play the more important role, as illustrated in Figure 12. Clearly, it is time for drought recovery to receive the scientific attention it deserves. The role of drought recovery should be further confirmed during whole growth period along with yield under field condition.

## Author contributions

SW and DC planned experiment. DC and SW conducted experiment, collected and analyzed the data, and prepared the draft. BC, DC, and GL helped measurements of physiological parameters. LY, HL, LS, and XD helped in drafting the manuscript and interpretation the results.

## Funding

This study was supported by Youth Innovation Promotion Association of the Chinese Academy of Sciences (2013307), National Key Technology Support Program of China (2015BAD22B01) and National Basic Research Program of China (2015CB150402).

### Conflict of interest statement

The authors declare that the research was conducted in the absence of any commercial or financial relationships that could be construed as a potential conflict of interest.
