# Supplementary material for: Genotypic Variation in Growth and Physiological Response to Drought Stress and Re-Watering Reveals the Critical Role of Recovery in Drought Adaptation in Maize Seedlings
Source: Front Plant Sci. 2016 Jan 12;6:1241. doi: 10.3389/fpls.2015.01241 (PMC4709455; doi:10.3389/fpls.2015.01241)
Supplement: Supplementary file 1 [file DataSheet1.pdf]

*Supplementary Material***:**

Genotypic variation in growth and physiological response to drought stress and re-watering reveals the critical role of recovery in drought adaptation in maize seedlings

Daoqian Chen, Shiwen Wang*, Beibei Cao, Dan Cao, Guohui Leng, Hongbing Li, Lina Yin , Lun Shan, Xiping Deng

* **Correspondence:**

Dr. Shiwen Wang

State Key Laboratory of Soil Erosion and Dryland Farming on the Loess Plateau

Institute of Soil and Water Conservation, Northwest A&F University

Xinong Road No. 26,

Yangling, Shaanxi, China. 712100.

shiwenwang@nwsuaf.edu.cn

Running title: Genotypic variation in maize drought adaptation

Supplementary Table

Supplementary TABLE 1 | Two-way ANOVA summary table for physiological traits of ten maize lines during drought stress and recovery. Degrees of freedom (d.f.) and F value are shown for each source of variation. * $P \leq 0.05$, ** $P \leq 0.01$, *** $P \leq 0.001$, ns, non-significant.

Parameter	Source of variation	d.f.	Drought		Recovery	
			F	Significance	F	Significance
RWC	Treatment	1	1613.7	***	17.7	***
	Lines	9	11.7	***	48.6	***
	T x L	9	11.4	***	11.3	***
WP	Treatment	1	3392.2	***	0.7	ns
	Lines	9	2.8	***	3.5	**
	T x L	9	2.8	**	2.9	**
OP	Treatment	1	2686.9	***	46.3	***
	Lines	9	33.1	***	48.7	***
	T x L	9	34.1	***	8.0	***
Pn	Treatment	1	114.6	***	16.7	***
	Lines	9	0.9	ns	7.2	***
	T x L	9	0.6	ns	3.1	**
Cond	Treatment	1	290.3	***	28.1	***
	Lines	9	1.2	ns	7.7	***
	T x L	9	0.9	ns	1.9	ns
Tri	Treatment	1	345.9	***	23.0	***
	Lines	9	1.3	ns	11.4	***
	T x L	9	1.5	ns	4.4	***
Chl	Treatment	1	205.1	***	0.4	ns
	Lines	9	12.9	***	9.1	***
	T x L	9	4.4	***	2.1	*
Car	Treatment	1	0.6	ns	6.1	*
	Lines	9	28.2	***	23.9	***
	T x L	9	11.0	***	1.7	ns
Fv/Fm	Treatment	1	227.7	***	1.3	ns
	Lines	9	3.8	**	2.3	*
	T x L	9	1.5	ns	1.3	ns
NPQ	Treatment	1	0.0	ns	7.5	**
	Lines	9	2.5	*	5.4	***
	T x L	9	1.2	ns	1.0	ns
YII	Treatment	1	115.3	***	3.5	ns
	Lines	9	5.9	***	4.8	***
	T x L	9	2.8	*	1.3	ns
H_2O_2	Treatment	1	85.9	***	26.3	***
	Lines	9	10.6	***	70.7	***
	T x L	9	3.1	**	15.9	***

MDA	Treatment	1	333.6	***	3.3	ns
	Lines	9	101.8	***	188.7	***
	T x L	9	50.1	***	19.9	***
Sugar	Treatment	1	0.0	ns	20.4	***
	Lines	9	20.0	***	128.8	***
	T x L	9	1.5	ns	5.1	***
Starch	Treatment	1	0.2	ns	3.4	ns
	Lines	9	14.1	***	26.7	***
	T x L	9	4.8	***	2.2	*
TNC	Treatment	1	0.4	ns	15.0	***
	Lines	9	10.1	***	27.3	***
	T x L	9	4.5	***	3.1	**
N	Treatment	1	3.9	ns	54.3	***
	Lines	9	24.3	***	43.1	***
	T x L	9	7.0	***	3.9	**
C/N	Treatment	1	2.2	ns	682.7	***
	Lines	9	520.0	***	810.2	***
	T x L	9	215.5	***	86.6	***

Supplementary Figure

Supplementary Figure 1. Changes in air temperature during seedling growth

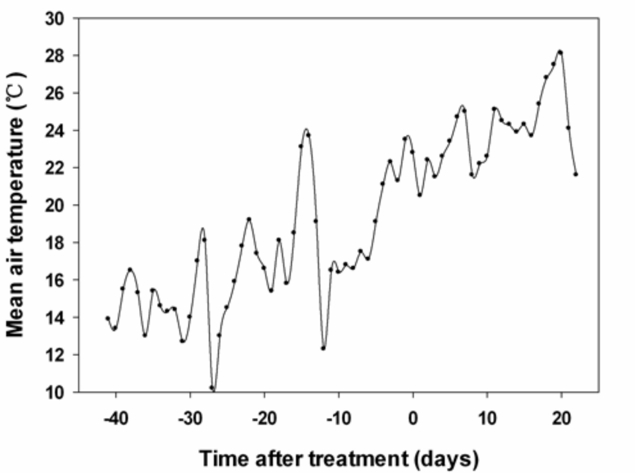

Supplementary FIGURE 1 | Changes in air temperature during seedling growth. Drought treatment started on day 0.
